# L-carnitine: a partner between immune response and lipid metabolism ?

**DOI:** 10.1155/S0962935193000729

**Published:** 1993

**Authors:** Giuseppe Famularo, Sonia Tzantzoglou, Gino Santini, Vito Trinchieri, Sonia Moretti, Aleardo Koverech, Claudio De Simone

**Affiliations:** 1 Infectious Diseases Via S. Sisto University of L'Aquila 67100 Italy; 2 Department of Internal Medicine and Public Health Via S. Sisto University of L'Aquila 67100 Italy; 3 Infectious Diseases University “La Sapienza” Rome Italy; 4 Sigma Tau Pomezia Italy

## Abstract

The authors demonstrated that *in vivo* administered L-carnitine strongly ameliorated the immune response in both healthy individuals receiving Intralipid and ageing subjects with cardiovascular diseases, as shown by the enhancement of mixed lymphocyte reaction. Notably, in the latter group L-carnitine treatment also resulted in a significant reduction of serum levels of both cholesterol and triglycerides. Therefore, the hypothesis is that L-carnitine supplementation could ameliorate both the dysregulated immune response and the abnormal lipid metabolism in several conditions.


Mediators of Inflammation 2, S29–S32 (1993)

### L-carnitine: a partner between immune response and lipid metabolism?

Giuseppe Famularo,[2,CA] Sonia Tzantzoglou,[3] Gino Santini,[3] Vito Trinchieri,[3] Sonia Moretti,[3] Aleardo Koverech,[4] and Claudio De Simone[1]

[1]Infectious Diseases and [2]Department of Internal Medicine and Public Health, Via S. Sisto, 67100, University of L'Aquila; [3]Infectious Diseases, University "La Sapienza" Rome; [4]Sigma Tau, Pomezia, Italy.

[CA] Corresponding Author

**THE authors demonstrated that *in vivo* administered L-carnitine strongly ameliorated the immune response in both healthy individuals receiving Intralipid and ageing subjects with cardiovascular diseases, as shown by the enhancement of mixed lymphocyte reaction. Notably, in the latter group L-carnitine treatment also resulted in a significant reduction of serum levels of both cholesterol and triglycerides. Therefore, the hypothesis is that L-carnitine supplementation could ameliorate both the dysregulated immune response and the abnormal lipid metabolism in several conditions.**

**Key words:** Immune response, L-carnitine, Lipid metabolism

### Introduction

Carnitine (3-hydroxy-4-methyl-ammoniobutanoate) is an essential intracellular constituent in higher animals which is synthesized from peptide bound lysine.[1,2] In meat and dairy products, carnitine is easily and almost completely absorbed, but endogenous biosynthesis can meet normal metabolic requirements of healthy adults.[3] Almost all the body stores are in skeletal and cardiac muscles (98%), whereas liver and extracellular fluids contain only 1–6% of whole-body carnitine.[4]

L-carnitine, the physiological isomer, is a non-toxic substance with an $LD_{50}$ similar to the $LD_{50}$ of amino acids. However, carnitine esters with long-chain acyls are significantly more toxic, as shown for example by palmityl carnitine exhibiting a 23-fold toxicity with respect to free carnitine.[5]

Carnitine and its derivatives have a key role in regulating substrate flux and energy balance across cell membranes.[2,6,7] When the carnitine dependent mechanisms are impaired fatty acid oxidation is reduced and triglycerides accumulate, therefore resulting into both microscopic fatty change[8] and impaired hepatic ketogenesis.[9] Carnitine is found in high concentrations in white blood cells.[10] However, the finding of most elevated carnitine concentrations in circulating mononuclear cells[10] first suggested the possibility that carnitine and its derivatives could modulate the function of immune cells and prompted us to assay the immunomodulant properties of carnitine both *in vitro* and *in vivo*.

### Materials and Methods

In vitro *studies:* L-carnitine was a gift from Sigma-Tau, Pomezia, Italy. Peripheral blood mononuclear cells (PBMCs) were obtained from humans, as described previously.[11,12]

For mitogen driven proliferative assays, PBMCs were resuspended at the concentration of $2 \times 10^6$ cells/ml in RPMI 1640 (Gibco Bio Cult., Paisley, UK) containing 10% heat-inactivated foetal calf serum (FCS), 1% L-glutamine, and penicillin/streptomycin. PBMCs were cultured for 72 h in a 5% $CO_2$ atmosphere at 37°C. Then, the cells were pulse-labelled with 1 $\mu$Ci of $^3$H-thymidine (Amersham, UK) in 20 $\mu$l medium 16 h before harvesting the cultures. The incorporation of $^3$H-thymidine was measured in a liquid scintillation beta counter.

In experiments carried out to evaluate the effects of Intralipid® (Laboratorien Hausmann AG, St Gallen, Switzerland) and Intralipid® plus L-carnitine were added at various concentrations to give a final volume of 60 $\mu$l at the beginning of the cultures.

For the determination of lactate dehydrogenase (LDH) isoenzyme patterns, PBMCs were incubated in a 5% $CO_2$ atmosphere at 37°C for 48 h in RPMI 1640 added with 10% FCS and antibiotics (control samples), with either Intralipid (100 $\mu$/ml) alone or Intralipid plus L-carnitine at various concentrations (1 and 10 $\mu$g/ml). Then, PBMCs were freeze–thawed three times and spun at 3 000 rpm for 10 min. The LDH isoenzymes of the supernatant fluid were determined by cellulose acetate electrophoresis

© 1993 Rapid Communications of Oxford Ltd of 5 μl samples for 30 min at 150 V. The areas of enzyme activity were then shown by histochemical procedures employing phenazine methosulphate and tetranitro blue tetrazolium in a sandwich technique. The electrophoretic pattern was scanned in a densitometer equipped with an integrator.

Ex vivo *studies:* Six healthy volunteers were first treated with Intralipid (20%) for 6 h. Heparinized (10 U/ml) peripheral venous blood was obtained by venipuncture before the infusion and then each hour up to the end of the administration of Intralipid. Peripheral venous blood was again obtained 24 h after the end of Intralipid infusion. The same subjects, after 2 days were treated with Intralipid plus L-carnitine (1 g/h) for the same time (6 h). Samples of heparinized venous blood were obtained as described above.

The ability of each plasma sample to inhibit mixed lymphocyte reaction (MLR) was tested. Briefly, purified and washed PBMCs, obtained as described above from healthy donors, were cultured in two-way mixed lymphocyte culture where for each time point triplicate measurements were performed. Mixtures contained 1 × $10^6$ cells from each of the two cell donors suspended in RPMI 1640 supplemented with antibiotics and 50% normal pooled plasma or plasmas (50%) obtained from the individuals treated with Intralipid and then with Intralipid plus L-carnitine. After 72 h of culture, PBMCs were pulse labelled with $^3$H-thymidine during the last 8 h of incubation. Then, the cells were collected into glass fibre filters and the radioactivity was measured in a beta counter. The degree of inhibition of the MLR, incubated with plasma obtained from volunteers treated with either Intralipid or with Intralipid plus L-carnitine, was expressed as a percentage of MLR incubated with plasmas collected before the administration of the drugs.

Ex vivo *and* in vivo *studies:* Twenty-three hospitalized patients, 15 males and eight females (mean age 75.5 ± 8 years) with cardiovascular diseases (cardiomyopathy, atroventricular block, atrial fibrillation, ventricular fibrillation, sick sinus syndrome) were enrolled into the study. The diagnosis was supported by both clinical criteria and non-invasive procedures (electrocardiography, phonocardiography, radiology and ultrasound).

The patients were treated with L-carnitine (3 g i.v. per day for 7 days). Peripheral blood samples were obtained at day 0 and at the end of the trial. Intradermal reactions to five recall antigens (candidine, parotitis, tricophytin, PPD and streptokinase–streptodornase) were tested by using the Multitest (Merieux Institute) immediately before and after treatment with L-carnitine.

**Table 1.** Effect of Intralipid® and Intralipid® plus L-carnitine on PHA-driven PBMC proliferation. The results are shown as mean cpm ± standard deviation of triplicate experiments

| Intralipid® (μl/ml) | L-carnitine (μg/ml) | | |
|---|---|---|---|
| | 0 | 1 | 10 |
| 0 | 57 062 ± 3 441 | 57 518 ± 2 883 | 58 623 ± 3 512 |
| 25 | 28 209 ± 2 994 | 33 542 ± 2 098 | 34 778 ± 2 741 |
| 50 | 27 362 ± 2 553 | 31 967 ± 2 531 | 38 678 ± 2 316* |
| 100 | 20 164 ± 1 537 | 24 250 ± 2 274 | 26 250 ± 1 883* |
| 250 | 4 564 ± 625 | 8 750 ± 933* | 9 893 ± 1 132* |

* $p < 0.01$

Total lipaemia was measured by the manual turbidimetric method with a linearity up to 1 000 mg/dl. Total cholesterol and triglycerides were measured with an Abbott-UP bichromatic analyser, by using a colorimetric enzymatic method and an enzymatic UV method, respectively, both with a linearity up to 500 mg/dl.

Serum carnitine was measured by the radioenzyme method described by McGarry and Foster,[14] with minor modifications as described previously.[15]

For the assays of MLR, either 50% normal pooled sera (from 60 healthy subjects) or sera from the patients enrolled into the study were added to the cell suspensions, using microtitre plates with flat-bottomed wells. The radioactivity measurement was performed as above described.

### Results and Discussion

The proliferation of mitogen (PHA) driven PBMCs from healthy individuals was strongly reduced by Intralipid (Table 1). At Intralipid concentrations up to 200 μl/ml, this inhibition was not due to cell death, as shown by PBMC samples tested for viability by trypan blue dye exclusion at different time points. Above this concentration, the cell viability was slightly impaired resulting in cell death of about 20% by the third day of culture (data not shown). Notably, the immunotoxic effects of Intralipid were dose-dependent. The addition of L-carnitine at various doses (1 and 10 μg/ml) exhibited a protective effect toward the immunotoxic action of Intralipid. Furthermore, the more marked the inhibition due to Intralipid the more marked was the restoring action of L-carnitine. In fact, at the highest Intralipid doses tested (250 μl/ml) L-carnitine increased the PBMCs incorporation of $^3$H-thymidine by about 20% ($p < 0.001$). The addition of L-carnitine at different doses indicated that the chance of finding doses effective under a statistically significant standpoint increased as the concentration of Intralipid in the medium was augmented. Overall, these data suggest that L-carnitine acts via a metabolic pathway more than by exerting a direct immunoenhancing effect and further evidence was found by performing the isoenzymatic analysis of LDH in PBMCs cultured for 48 h. Both control PBMCs and PBMCs treated with Intralipid plus L-carnitine contained more LDH1 isoenzyme and more heart sub-units compared to PBMCs treated with Intralipid alone ($p < 0.01$). This finding possibly reflected a greater aerobic glycolysis, as the LDH1 tissue content has been considered to be proportional to the degree of aerobic metabolism. Likewise, the LDH pattern of PBMCs treated with Intralipid alone contained more LDH5, likely pointing out the anaerobic metabolism of cells cultured in the presence of the fat emulsion.

In studying *ex vivo* the effects of Intralipid and L-carnitine in healthy individuals, a modification of the two-way MLR for the assessment of the immunosuppressive activity was employed. The challenge of plasmas from subjects receiving Intralipid with lymphocytes from healthy unrelated donors resulted in a mean 9% inhibition of the lymphocyte reaction after 1 h from the start of the infusion. The administration of L-carnitine in conjunction with Intralipid resulted, conversely, in an increase of the responses (mean 12% after 60 min). Therefore, it resulted that after the first hour of infusion the total difference between plasmas from subjects treated with Intralipid and subjects treated with Intralipid plus L-carnitine was about 21% when tested for immunosuppressive activity of MLR. This difference decreased to 7% after 24 h, probably due to host's adaptative metabolic mechanisms.

Overall, the *in vitro* and *in vivo* studies pointed out that the immunotoxic action of Intralipid could be reversed, at least to a certain degree, by adding L-carnitine to PBMC cultures, even at minimal concentrations. Since a direct immunoenhancing effect of L-carnitine was ruled out, in the authors' opinion the most probable hypothesis is that L-carnitine could restore the immune response by removing some metabolic cofactor limitation of PBMCs cultured in the presence of Intralipid.

The treatment of elderly patients suffering from cardiovascular diseases with L-carnitine (3 g per day for 7 days) resulted in a significant reduction of lipaemia, cholesterolaemia, and triglyceridaemia in the majority of cases (Table 2). Furthermore, this result was paralleled by both an increase, *in vivo*, in the delayed hypersensitivity response to common recall antigens (candidine, tricophytin, parotitis, PPD, streptokinase–streptodornase) and in the ability of serum to stimulate MLR (Table 3). Notably, skin test reactivity increased in the same patients in whom a reduction of serum lipid levels was observed following L-carnitine treatment. Similarly, MLR was increased when the cells were

**Table 2.** Serum levels of cholesterol (normal range: 160–330 mg/dl) and triglycerides (normal range: 40–160 mg/dl) before (A) and after (B) L-carnitine supplementation in ageing patients with cardiovascular diseases

| Patient | Cholesterol | | Triglycerides | |
|---|---|---|---|---|
| | A | B | A | B |
| 1 | 231 | 212 | 138 | 130 |
| 2 | 279 | 235 | 168 | 145 |
| 3 | 288 | 231 | 171 | 164 |
| 4 | 197 | 199 | 143 | 144 |
| 5 | 281 | 243 | 177 | 169 |
| 6 | 121 | 156 | 168 | 112 |
| 7 | 200 | 203 | 136 | 135 |
| 8 | 157 | 150 | 140 | 114 |
| 9 | 181 | 251 | 184 | 281 |
| 10 | 191 | 155 | 112 | 109 |
| 11 | 231 | 196 | 117 | 117 |
| 12 | 163 | 175 | 75 | 83 |
| 13 | 199 | 164 | 114 | 108 |
| 14 | 175 | 180 | 106 | 106 |
| 15 | 269 | 198 | 134 | 132 |
| 16 | 215 | 187 | 176 | 122 |
| 17 | 152 | 136 | 163 | 124 |
| 18 | 175 | 110 | 116 | 113 |
| 19 | 230 | 242 | 114 | 126 |
| 20 | 176 | 183 | 98 | 106 |
| 21 | 157 | 136 | 87 | 85 |
| 22 | 196 | 177 | 126 | 109 |
| 23 | 226 | 185 | 130 | 130 |

**Table 3.** Delayed hypersensitivity skin test and mixed lymphocyte reactions (MLR) before (A) and after (B) L-carnitine supplementation in ageing patients with cardiovascular diseases

| Patient | Skin tests | | MLR |
|---|---|---|---|
| | A | B | % variation |
| 1 | + | +++ | +14 |
| 2 | + | ++ | +19 |
| 3 | +++ | ++++ | +12 |
| 4 | ++ | ++ | −3 |
| 5 | + | ++ | +21 |
| 6 | + | +++ | +35 |
| 7 | +++ | ++ | 0 |
| 8 | ++ | +++ | +16 |
| 9 | − | − | −12 |
| 10 | + | ++ | +9 |
| 11 | − | + | +23 |
| 12 | − | − | 0 |
| 13 | ++ | ++ | +23 |
| 14 | + | − | −17 |
| 15 | − | + | −6 |
| 16 | − | ++ | +22 |
| 17 | + | ++ | +16 |
| 18 | + | ++ | +14 |
| 19 | − | − | 0 |
| 20 | − | − | 0 |
| 21 | − | + | +18 |
| 22 | − | + | +12 |
| 23 | + | ++ | +10 |

+ = ⩾5 mm ⩽ 10 mm; ++ = ⩾10 mm ⩽ 15 mm; +++ = ⩾15 mm.

added to or preincubated with post-treatment sera obtained from the same patients, compared to the results obtained by using the pre-treatment sera. Patients exhibiting no effect to L-carnitine administration in lowering blood lipid levels also had unmodified or even reduced skin test reactivity to recall antigens. Their sera were also unable to increase MLR. The general trend in this study was towards a lowering of lipaemia, cholesterolaemia, and triglyceridaemia and in enhanced host immune responsiveness following L-carnitine supplementation. The possibility that L-carnitine had a direct immunoenhancing property was ruled out by finding that high levels of serum L-carnitine were associated with unchanged immunological parameters in patients exhibiting no reduction of blood lipids following L-carnitine treatment.

In conclusion, the above experiments suggest a direct relationship between L-carnitine, lipids and immune responsiveness. The mechanisms accounting for the effects of L-carnitine on human lymphocytes in the presence of a lipidic dysmetabolism have yet to be fully established.

### References

1. Rebouche CJ, Engel AG. Tissue distribution of carnitine biosynthetic enzymes in man. *Biochim Biophys Acta* 1980; **630**: 22–29.
2. Siliprandi N, Di Lisa F, Menabo R, Ciman M, Sartorelli L. Transport and functions of carnitine in muscles. *J Clin Chem Clin Biochem* 1990; **28**: 303–306.
3. Feller AG, Rudman D. Role of carnitine in human nutrition. *J Nutr* 1988; **118**: 541–547.
4. Engel AG, Rebouche CJ. Carnitine metabolism and inborn errors. *J Inherited Metab Dis* 1984; **7 (suppl. 1)**: 38–43.
5. Haeckel R, Kaiser E, Oellerich M, Siliprandi N. Carnitine. Metabolism, function and clinical application. *J Clin Chem Clin Biochem* 1990; **28**: 291–295.
6. Bremer J. The role of carnitine in intracellular metabolism. *J Clin Chem Clin Biochem* 1990; **28**: 297–301.
7. Bremer J. Carnitine: metabolism and function. *Physiol Rev* 1983; **63**: 1420–1480.
8. Gilbert EF. Carnitine deficiency. *Pathology* 1985; **17**: 161–169.
9. Chapoy PR, Angelini C, Brown WJ, Stiff JE, Shug AL, Cederbaum SD. Systemic carnitine deficiency: a treatable inherited lipid-storage disorder presenting as a Reye's syndrome. *N Engl J Med* 1980; **303**: 1389–1394.
10. Deufel T. Determination of L-carnitine in biological fluids and tissues. *J Clin Chem Clin Biochem* 1990; **28**: 307–311.
11. Famularo G, Giacomelli R, Valdarnini D, De Simone C, Tonietti G. Modulation by ST 789 of *in vitro* lymphocyte activation and cytokine production. *Thymus* 1992; **19 (suppl. 1)**: 571–578.
12. De Simone C, Arrigoni Martelli E, Foresta P, *et al.* Hypoxanthine derivatives in experimental infections. *Cand J Infect Dis* 1992; **3 (suppl. B)**: 106–110.
13. Famularo G, Giacomelli R, Di Giovanni S, Sacchetti S, Tonietti G. Cytokine production in patients with monoclonal gammapathies. *J Clin Lab Immunol* 1991; **34**: 63–70.
14. McGarry JD, Foster DW. Free and esterified carnitine: radio method. In: Bergmeyer J, Grassl MW, eds. *Methods of Enzymatic Analysis.* Berlin: Verlag, 1983; 474–481.
